# Environmental Conditions Affecting *Ochratoxin A* during Solar Drying of Grapes: The Case of Tunnel and Open Air-Drying

**DOI:** 10.3390/toxins13060400

**Published:** 2021-06-03

**Authors:** Charalampos Templalexis, Paola Giorni, Diamanto Lentzou, Sabrina Mesisca, Dimitrios I. Tsitsigiannis, Paola Battilani, Georgios Xanthopoulos

**Affiliations:** 1 Department of Natural Resources Management and Agricultural Engineering, Agricultural University of Athens, 75 Iera Odos Str., 11855 Athens, Greece; chartempl@aua.gr (C.T.); dlen@aua.gr (D.L.); 2 Department of Sustainable Crop Production (DI.PRO.VE.S.), Università Cattolica del Sacro Cuore, Via Emilia Parmense 84, 29122 Piacenza, Italy; paola.giorni@unicatt.it (P.G.); sabrina.mesisca@unicatt.it (S.M.); paola.battilani@unicatt.it (P.B.); 3 Department of Crop Science, Agricultural University of Athens, 75 Iera Odos Str., 11855 Athens, Greece; dimtsi@aua.gr

**Keywords:** *Aspergillus carbonarious*, *Ochratoxin A*, open air–drying, tunnel drying, water diffusivity, grapes, water surface resistance

## Abstract

Drying optimization, to mitigate fungal growth and *Ochratoxin A* (OTA) contamination is a key topic for raisin and currant production. Specific indicators of environmental conditions and drying properties were analyzed using two seedless grape varieties (*Crimson*—red and *Thompson*—white), artificially inoculated with *Aspergillus carbonarius* under open air and tunnel drying. The air temperature (T), relative humidity, grape surface temperature (T_s_) and water activity throughout the drying experiment, the grapes’ moisture content and the fungal colonization and OTA contamination during the drying process and their interactions were recorded and critically analyzed. Drying properties such as the water diffusivity (D_eff_) and peel resistance to water transfer were estimated. The grapes T_s_ was 5–7 °C higher in tunnel vs. open air–drying; the infected grapes had higher maximum T_s_ vs. the control (around 4–6 °C). OTA contamination was higher in tunnel vs. open air–dried grapes, but fungal colonies showed the opposite trend. The D_eff_ was higher in tunnel than in the open air–drying by 54%; the infected grapes had more than 70% higher D_eff_ than the control, differences explained by factors affecting the water transport. This study highlighted CFU and OTA indicators that affect the water availability between red and white grapes during open air and tunnel drying, estimated by the D_eff_ and peel resistance. This raises new issues for future research.

## 1. Introduction

Grapes are susceptible to insect attack and fungal diseases, especially black and grey rot, downy and powdery mildew. Damaged grapes are vulnerable to further diseases such as summer bunch rot, which may be caused by *Aspergillus niger*, *Alternaria tenuis*, *Cladosporium herbarum*, *Rhizopus arrhizus*, *Penicillium spp.* and other fungi. Fungal invasion depends on grape maturity. *Alternaria*, *Cladosporium*, *Botrytis* and *Rhizopus* are common at early veraison whereas *Aspergillus* and *Penicillium* are frequently detected at harvest and during sun drying. *Aspergillus carbonarius* and *Aspergillus niger* are known to be responsible for *Ochratoxin A* (OTA) contamination in grapes and raisins [1,2,3,4]. The critical factors that affect fungal growth during farming, harvesting and storage are temperature, moisture content (water activity—a_w_) and time that product remains under favorable conditions for fungi (integral of time–temperature–water activity). Other factors are the presence of fungal spores, mechanical damages (the inner area in vegetables is more susceptible to fungal invasion than the external due to lack of protective peel), insect with piercing–sucking mouthparts (with their metabolism increase substrate’s moisture and temperature and break the protective external part of the plant), storm and rain damages, moisture stress, mineral nutrients deficiency, pH, O_2_ and CO_2_ levels, chemical and physical treatments and for some commodities, the product drying and re–wetting speed. Nonetheless, it is important to note that molds presence does not imply OTA production, as this is triggered by certain conditions of temperature, moisture, O_2_, time or nutrient sufficiency. Thus, it is possible to detect OTA in grape even without visible signs of the producing fungi presence [1]. The region of grape origin can greatly influence OTA contamination showing a positive gradient moving from west to east and from north to south in Europe [5].

Mehri et al. [6] conducting an exhaustive review concerning the production of OTA in dried grapes, concluded that critical factors such as physicochemical properties (ratio of water, dry matter, sugars, acids, flavonoids, pH, etc.), management condition (pre– and post–harvest, storage and processing conditions) and weather conditions (ratio of humidity, environmental temperature, and rainfall) determine fungal growth and lead to differences in the prevalence and concentration of OTA in grape based products. In particular, *A. carbonarius*, the most efficient OTA producer, becomes predominant in respect to *A. niger* during the drying process since it is more adaptable to a low a_w_ compared to other black *Aspergilli* [7]. The ecological parameters of black *Aspergilli* were largely studied, and this knowledge is critical in the development of risk models of grapes and grape products contamination by this species under fluctuating and interacting environmental parameters [8].

*A. carbonarius* has been reported as the main source of OTA contamination in wine and in dried vine fruits. Dried grapes are healthy foods and are ingredients in muesli, cereal bars, biscuits and cakes, among other foods, and could be an important source of OTA for those who consume large amounts, particularly children. The Commission Regulation (EC) No 1881/2006 has established the maximum OTA content of 10 μg/kg for these products (currants, raisins and sultanas). *A. carbonarius* and *A. niger* produce single–celled conidia with melanin and *aspergilline* in their cell walls, but differ in their UVC resistance and their incidence on grapes [9]. *A. carbonarius* spores are characterized by thicker walls than the 90–160 nm of *A. niger*. The higher UVC resistance of *A. carbonarius* spores compared to *A. niger* spore, provides a logical explanation for the high incidence of *A. carbonarius* on grapes subjected to prolonged sun exposure. *Aspergillus* section *Nigri spp.* was found in more than 80% of sun–dried grapes [3]. In particular, *A. carbonarius* occurred in increased amount compared with *A. niger* on raisins and dried vine fruits [10,11,12].

This study aims to analyze important aspects of grape open air and tunnel solar drying. The analysis of the critical points (drying temperature, air humidity, a_w_ and drying time), drying properties (water diffusivity (D_eff_) and peel resistance to water transport (r_peel_)) and colony forming units (CFU) and OTA production, will derive useful conclusions regarding their influence on the two widely used drying methods. For the first time the resistance of grape peel to water transport will be also evaluated and associated with the fungal colonization (CFU) and OTA production during solar drying.

## 2. Results and Discussion

Based on the conducted measurements, the geometric average, min and max values were estimated (Table 1). The surface temperature of the grape berries was 5–7 °C on average higher in tunnel drying grape berries than in open air–drying grape berries. Interestingly, the infected grape berries had higher maximum surface temperatures compared to the control grape berries (approximately 4–6 °C) especially in the case of open air–drying (Table 1).

**Figure 1:**
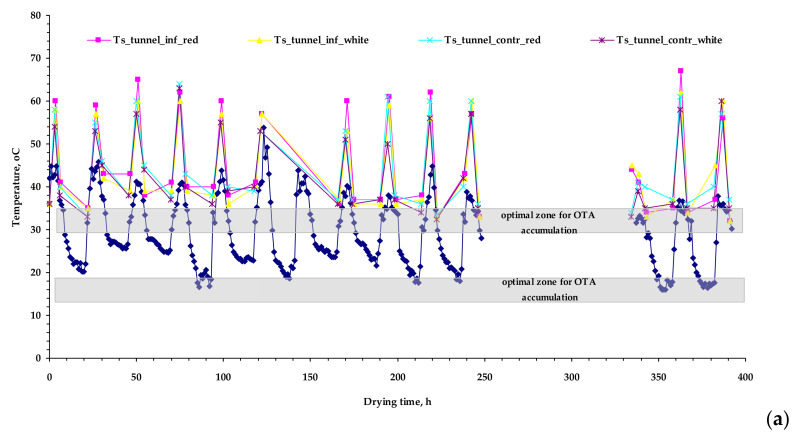

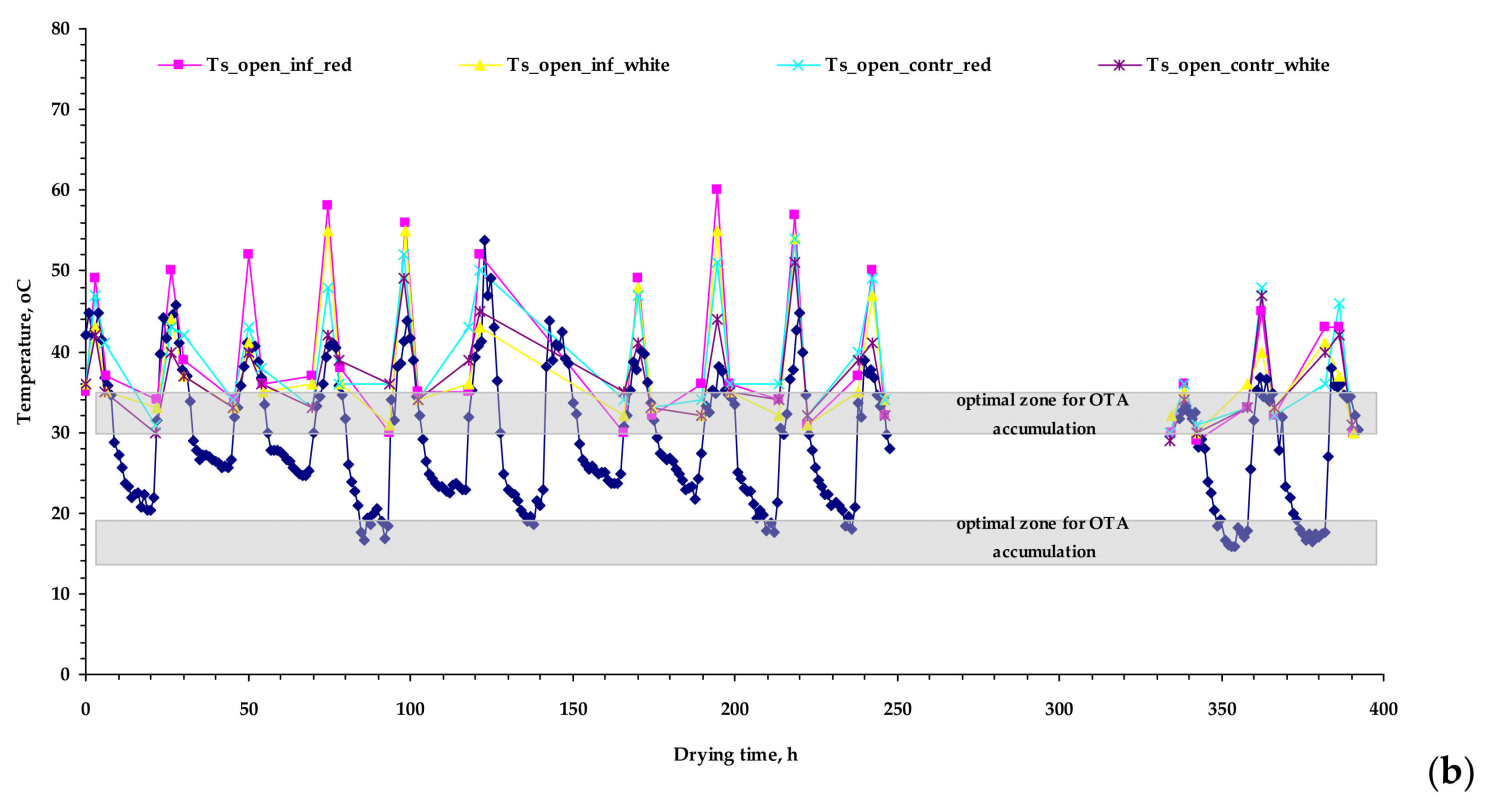
Air temperature (blue) during the experimental period (07 September 2020–23 September 2020) and surface temperature (T_s_) of infected and control red and white grapes [tunnel drying (**a**) and open air–drying (**b**)] during the daily measurement period (09.00–18.30).

This temperature pattern has been also identified by thermal imagery between healthy and artificially infected table grapes (var. *Crimson*) [13]. This response can be seen in Figure 1 where the surface temperature in all the grape berries is more than 10 °C higher than the respective air temperature. The grape berries’ temperature is an important factor along with a_w_ in fungal infection, as in the absence of surface wounds infection spreads on the surface of the grape berries [14,15].

To the best of our knowledge, this is the first time where along with the air temperature, the temperature of the grape surface is also investigated during grape solar air–drying. Statistical analysis of the air temperature and the surface temperatures in the eight drying experimental treatments seen in Table 1, allocate the temperatures in four homogenous groups identified using columns of “X”. Within each column, the levels containing “X” form a group of means within which there are no statistically significant differences. Identification of the temperature mean difference per experimental treatment is based on Tukey’s multiple comparison procedure (*p* ≤ 0.05). Therefore, significant difference exists between the air temperature and the surface temperatures, and hence the grape berry surface temperature should be taken into consideration when OTA production is investigated during drying of agricultural products because it can significantly influence the OTA production.

**Figure 2:**
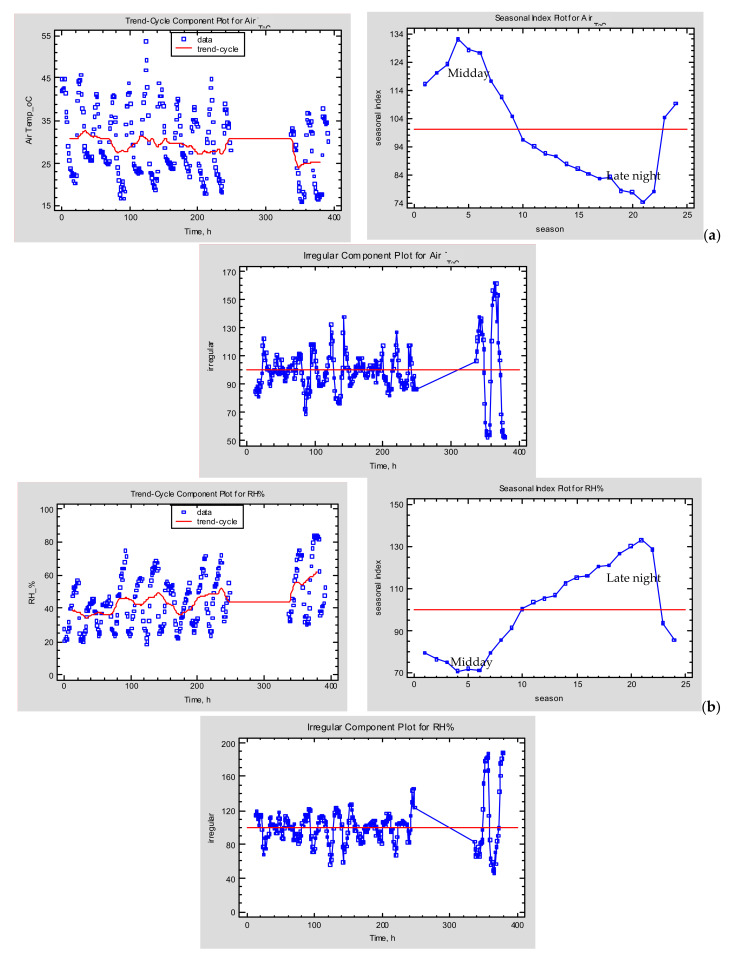
Seasonal decomposition plots (trend–cycle, seasonality (1–24 h), irregular) for the air temperature (**a**) and the air relative humidity (RH%) (**b**) during the drying period (7 September 2020–23 September 2020).

The seasonal indices (season = 1 h) for air temperature (Figure 2a) range from a low of 74.16 at hour 21, to a high of 132.05 at hour 4, indicating that there is a seasonal variation from 74.16% of average (20.8 °C) to 132.05% of average (37.10 °C) throughout the course of a complete cycle (day). Similarly, the seasonal indices (season = 1 h) of relative humidity (RH) (Figure 2b) range from a low of 70.89% at hour 4 to a high of 133.13% at hour 21. This indicates that there is a seasonal variation from 70.89% of average (29.63%) to 133.13% of average (55.65%) throughout the course of a complete cycle. The results from the seasonal decomposition compared to the environmental data in Table 1, reveal that the air temperature and relative humidity exhibit a simulation fuzziness which can be source of potential error in the prediction of fungal proliferation and OTA accumulation during the drying process in open air. The choice of the appropriate data input regarding the air temperature and humidity in prediction models such as Decision Support Systems (DSS), for the early warning prediction of *A. carbonarius* proliferation and OTA production in table and wine making grape varieties is of outmost importance.

Environmental conditions are important for black *Aspergilli* growth and OTA production, mainly air temperature, rainfall and relative humidity. *A. carbonarius* grows optimally at 30–35 °C, and the optimal a_w_ reported varied from 0.92–0.98 with the widest range at 25–35 °C [16]. Regarding OTA production, optimum conditions were stated at a_w_ = 0.95–0.98 and air temperatures of 15–20 °C or 30–35 °C, depending on the strains, but irrespective of their geographical origin [8,16].

Thus, apart from the true temperature of the drying grapes, it is very important to model as well, the a_w_ of grapes during drying. For this purpose, the well–known model of G.A.B. sorption isotherm was used. The M_o_, C and K_b_ were estimated by the Levenberg–Marquardt optimization algorithm (Table 2), where M_o_ is the monolayer moisture content; C and K are the adsorption constants.

**Figure 3:**
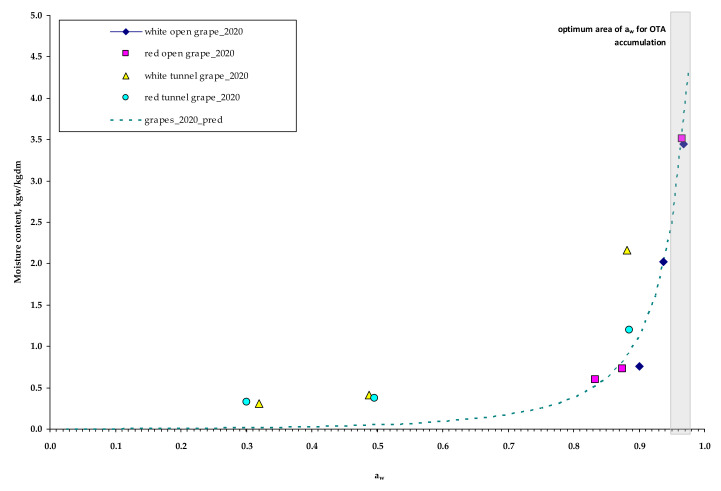
Water activity of drying grapes (white and red) during the solar drying experiments (open–air and tunnel) conducted in September 2020. (“*pred*” stands for predicted).

The statistical analysis gave $R_{\mathrm{adj}}^{2}=$ 96.31% and SEE = 0.2277 (*p* ≤ 0.05). The experimental (points) and predicted (line) a_w_ values are presented in Figure 3.

Based on the experimental data at the middle sampling date in 14 September 2020, the estimated a_w_ was lower than 0.9 in the red and white grapes dried in tunnel, but not in the respective open air–drying grapes, where the drying process lasted much longer. Nevertheless, OTA contamination was higher in the tunnel compared to the open air–dried grapes, but CFU/mL showed the opposite trend (Table 3).

The drying data from all the experimental treatments (tunnel and open air–drying, infected and control red and white grapes) were analyzed to estimate their drying properties by computational simulation of the drying process per drying case as this was presented by Lentzou et al. [17]. The drying samples used in weighing (20 grape berries per drying case) were daily photographed and the digital photos were processed by the Adobe Photoshop, v.13012 (Adobe Photoshop inc. USA) to evaluate the shrinkage effect.

**Figure 4:**
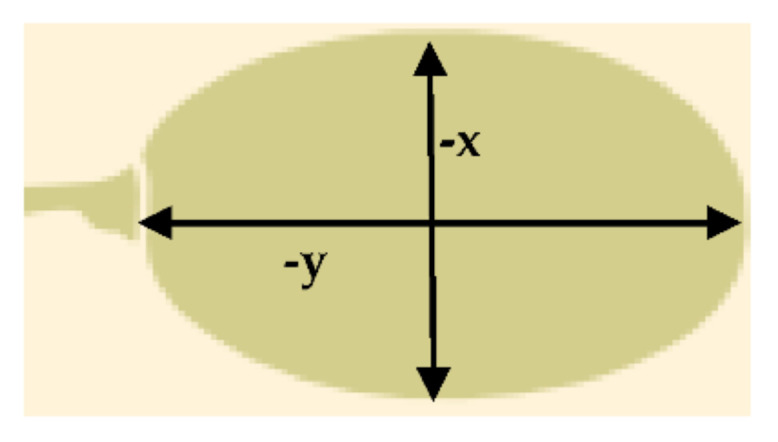
Grape berries were considered as prolate spheroids during computational simulation.

The grape berries were considered as prolate spheroids having two axes, a major (y) and a minor (x) which were reduced asymmetrically as drying was progressed (Figure 4). From image analysis, the shrinkage velocity (*m*/*s*) per drying case was estimated, tabulated in Table 4 and fed to computational model as input data.

**Figure 5:**
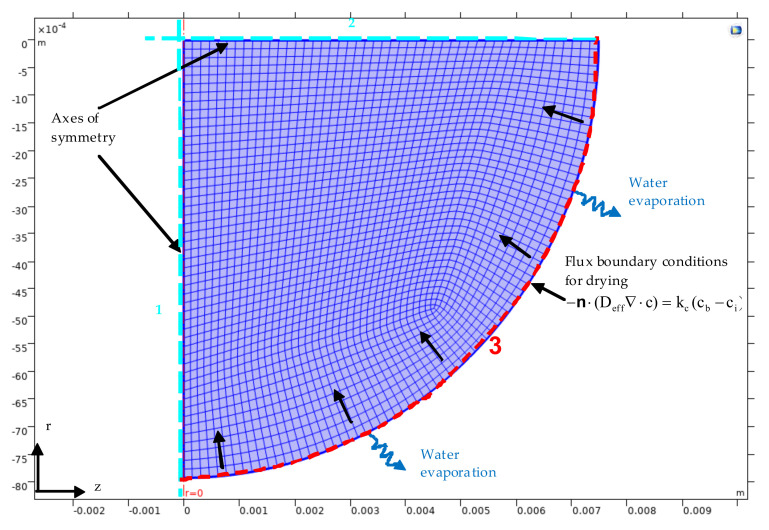
Geometry discretization of the (shrinking) domain and boundary conditions; two axial symmetry conditions (1, 2) and boundary condition (3).

The physical problem under consideration gives rise to a computational model for deriving theoretical predictions of the spatiotemporal distribution of water content in drying whole grape berries as function of the effective water diffusivity (D_eff_), peel resistance to water transfer (r_peel_) and shrinkage. The governing equations along with the boundary and initial conditions were numerically discretized by the Finite Element Method (FEM) using COMSOL Multiphysics 5.1. The unstructured mesh composed of approximately 2350 free quad elements (Figure 5).

In the present study, the D_eff_ and the surface mass transfer coefficient (k_c_) were estimated solving the *Fick’s* law of diffusion problem employing the experimental drying curves [MC = *f*(t)]. For this reason, the Levenberg–Marquardt optimization algorithm and the finite element method were combined to estimate the two coefficients (D_eff_, k_c_) in an inverse mass transfer problem taking place during drying of grape berries with shrinkage. The time–dependent problem was solved by the Backward Differentiation Formula (BDF) which belongs to a family of implicit time stepping methods for the numerical integration of ordinary differential equations. The resulting system of nonlinear PDEs (Partial Differential Equation) in the time–space domain was solved numerically by coupling the FEM with the Arbitrary Lagrange–Eulerian (ALE) procedure to account for the shrinkage. In the ALE method, the boundary conditions (Table 1 and Table 4) control the displacement of the moving mesh with respect to the initial geometry. The moving boundary displacement is propagated throughout the domain to obtain a mesh deformation everywhere using a Laplace smoothing technique. At each iteration step, the linearized equation set was solved with the Parallel Sparse Direct Solver (PARDISO). This solver is faster than the other available linear solvers. As time step, was set the value of one hour even though the COMSOL Multiphysics 5.1 implements an internal variable time step to satisfy the conditions of the relative (R_tol_) and absolute tolerance (A_tol_) which were set to 0.0001 and 0.00001, respectively. Solutions reliability was ensured by grid independency tests. Inverse problems are ill–posed having multiple possible solutions rather than a unique solution. This makes their solution prone to errors if the minimization of the objective function is only considered. Therefore, three criteria were adopted, to enforce the choice of the optimization parameter values:

- The D_eff_ should range 10^–11^–10^–9^ m^2^/s where most of the foodstuffs D_eff_ have been reported [18].
- The predicted D_eff_ should be less than the respective for self–diffusion of water [3.6 × 10^−9^ m^2^/s (at 45 °C), 4.37 × 10^−9^ m^2^/s (at 55 °C) and 5.09×10^−9^ m^2^/s (at 65 °C)] [17].
- The mean relative error (MRE) between the estimated and the experimental water content should be below 10%.

**Figure 6:**
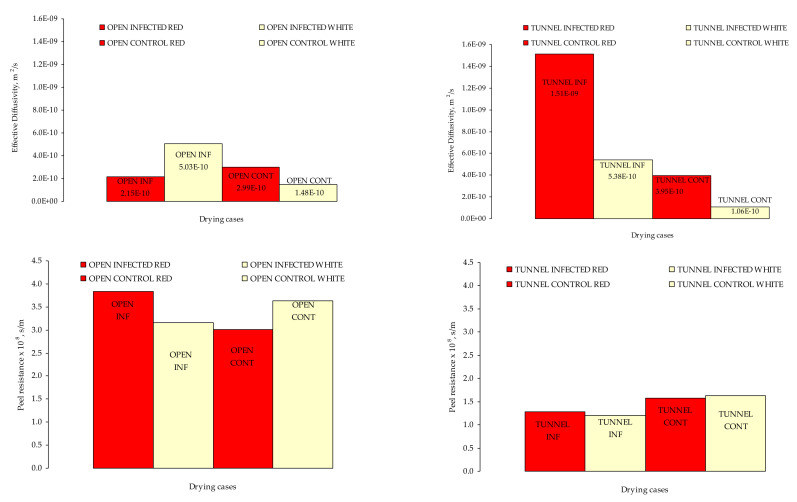
The D_eff_ (m^2^/s) and the r_peel_ (s/m) of red and white grapes per tested drying case.

The optimization procedure was based on input values tabulated in Table 1. These values are characteristic for the drying period as this was previously discussed within the daytime. The visualization of the estimated properties k_c_, mean D_eff_ and surface resistance to water transport (r_peel_ = 1/k_c_) for all the drying cases (Table 5) are presented in Figure 6.

As can be seen, the D_eff_ in tunnel drying was higher than the respective in the open air–drying by 54% on average. Partially, this can be explained from the temperature difference which was on average 6 °C higher in the tunnel than in the open air–drying. Comparing the D_eff_ between infected and control samples per drying case, it can be seen that in tunnel drying, the infected grape berries had more than 70% higher D_eff_ than the respective control grape berries (red grapes: 74%, white grapes: 80%). The specific finding is of great importance since the infected and control, tunnel dried grape berries were dried under the same temperature on average (Table 1) and therefore the D_eff_ difference can be explained only by factors affecting the water transport. The water transport mechanisms are complex since the drying rate in this case is affected by the biology/anatomy of the grape berry, peel resistance to water transfer of each grape variety and stage of maturity. The previous results are in line with the fact that the *Aspergillus* growth is favored the red grapes compared to the white ones. The skin of the grape berries is covered by a waxy layer (cuticle) and has only a small number of functional stomata, thus water loss occurs mostly through the waxy cuticle at a relatively slow rate. If the rate of water loss increases due to high temperatures, skin splitting is caused. When the skin is damaged, nutrients are no longer limiting, and the microbial population increases dramatically. Skin damage can be caused by many factors, including disease (black spot, downy mildew, powdery mildew), pests (bunch, mites, mealy bug, light brown apple moth) and the vineyard environment (wind injury, sunburn, hail damage, bird damage) [14]. Some of the previous factors cause localized hardening of the skin, which may increase the susceptibility of the fruit to splitting [14]. Zhang et al. [19] investigated the effect of dipping pre–treatment on OTA accumulation in sultanas (white grapes) and currants (red grapes) and concluded that in the untreated samples OTA accumulation was more serious in red grapes than in white grapes, and that in particular this behavior was attributed to the components of red dried grapes that may favor fungal growth, which lead to a faster and higher accumulation of OTA.

The estimated r_peel_ exhibited a distinctive behavior during drying per tested treatment. The r_peel_ of tunnel drying grapes was ≈2.4 times lower than the respective in open air–drying grapes (Table 5). This finding is in line with the drying time which was approximately 390 h at the open air–drying case and 220 h at the tunnel drying considering that drying is ended when no mass reduction is noted. In tunnel drying, the r_peel_ in white control grape berries was 35% higher than the respective in white infected and the red control grape berries was 22% higher than in the respective red infected (Figure 6).

**Figure 7:**
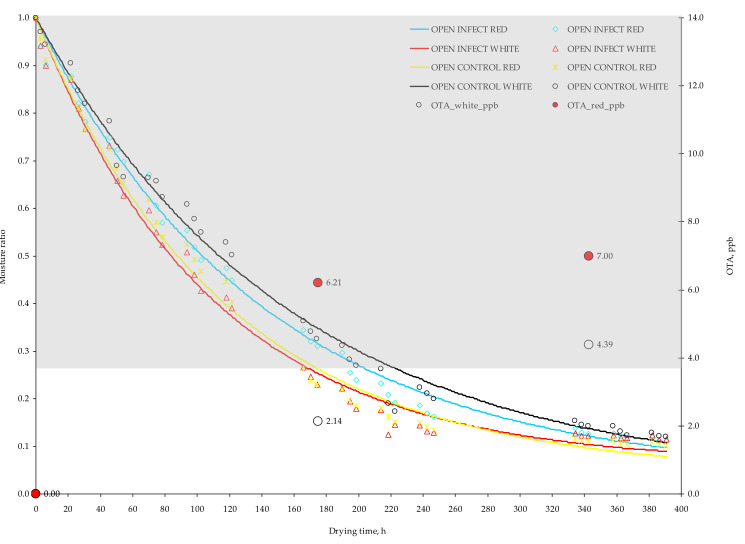
Experimental (points) and predicted (lines) moisture ratios of red and white grapes subjected in open air–drying. The red and white points are the experimental accumulated OTA (ppb) in red and white grape berries respectively. Grey highlighted area represents the optimum zone (MR = 1.0, a_w_ = 0.98; MR = 0.27, a_w_ = 0.92) for *A. carbonarius* growth based on Equation (1). Infected: ${\mathrm{MC}}_{o}^{\mathrm{red}}=$ 4.46kg_water_/kg_dm_, ${\mathrm{MC}}_{o}^{white}=$ 4.90kg_water_/kg_dm_; Control: ${\mathrm{MC}}_{o}^{\mathrm{red}}=$ 4.84kg_water_/kg_dm_, ${\mathrm{MC}}_{o}^{white}=$ 3.82kg_water_/kg_dm_.

**Figure 8:**
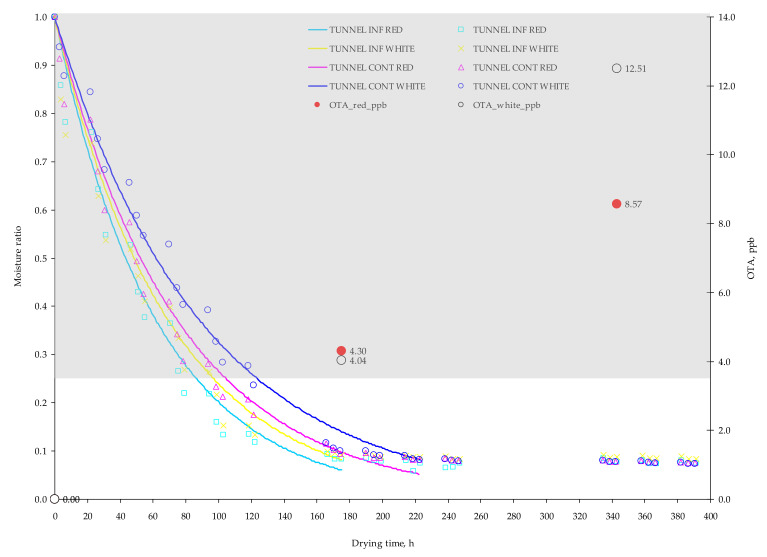
Experimental (points) and predicted (lines) moisture ratios of red and white grapes subjected in tunnel drying. The red and white points are the experimental accumulated OTA (ppb) in red and white grape berries respectively. Grey highlighted area represents the optimum zone (MR = 1.0, a_w_ = 0.98; MR = 0.27, a_w_ = 0.92) for *A. carbonarius* growth based on Equation (1). Infected: ${\mathrm{MC}}_{o}^{\mathrm{red}}=$ 4.07kg_water_/kg_dm_, ${\mathrm{MC}}_{o}^{white}=$ 4.23kg_water_/kg_dm_; Control: ${\mathrm{MC}}_{o}^{\mathrm{red}}=$ 4.27kg_water_/kg_dm_, ${\mathrm{MC}}_{o}^{white}=$ 4.08kg_water_/kg_dm_.

Lentzou et al. [17] attributed the previous response to disintegration of the peel or other structural changes taking place during drying such as berry flesh collapsing which might cause reduction of the pathway to the surface and therefore reduction of the peel resistance. As can be seen from Table 1, the mean drying temperature in the tunnel drying grapes (≈45 °C) was 18% higher than in the open drying grapes (≈38 °C). To enhance the water transport through grape skin and reduce the respective drying time, the chemical pre–treatment of grapes is suggested. On the other hand, the chemical pre–treatment in grapes has been reported to increase the potential hazard of OTA development especially in dried grapes stored unpackaged after drying [19]. The drying temperatures in the present solar drying experiments were lower compared to artificial drying temperatures (>50 °C) normally employed in drying of agricultural products. The previous results are based on computational simulations and therefore they need to be validated by an electronic microscopy analysis of peel disintegration during the solar drying process. The image analysis from the electronic microscopy can reveal the potential peel disintegration in the control and infected grapes (red and white) regarding the employing drying method as well as the degree of effect on the disintegration of the grape peel by the *Aspergillus* growth and OTA development. The computational simulation managed to predict efficiently the experimental moisture content having MRE less than 10% (Table 5). These findings can be seen in Figure 7 and Figure 8 where the drying curves [MR = *f*(t)] along with the production of OTA are illustrated. The grey highlighted areas represent the optimum zone (MR = 1.0, a_w_ = 0.98; MR = 0.27, a_w_ = 0.92) for *A. carbonarius* growth based on Equation (1).

Contrary, considering fungal infection, despite the estimated higher r_peel_ (Table 5), ANOVA underlined a higher CFU/mL in open air–drying compared to tunnel–drying conditions (*p* ≤ 0.01) (Table 3). This was probably due to (i) the longer drying time in open air, (170 h longer than tunnel drying) and, consequently, to more time in which fungi can continue to develop and sporulate, and (ii) the more optimum temperature conditions for fungal growth on grape berries (Table 1). The importance of drying duration on product safety has been already confirmed on other types of foods, for example dried figs where the low drying rate of the drying process in open air, depending mainly on the climatic conditions (air temperature and humidity, solar radiation), can result in diverse problems such as the rapid growth and proliferation of microorganisms so as mycotoxin production [20].

Considering OTA production, open air–drying showed a lower content in comparison with tunnel drying. This can be the result of the faster reduction in a_w_ content of grapes berries under tunnel conditions, that promoted a_w_ × temperature stress able to favor OTA production in the presence of black *Aspergilli* population (Table 3 and Figure 7 and Figure 8). Previous studies highlighted the impact that single and interacting environmental stress factors have on the relationship with secondary metabolite production by mycotoxigenic species; in particular, the genes for toxin production are clustered together and their relative expression is influenced by abiotic interacting stress factors like marginal temperature and a_w_ regimes [21,22,23].

Red grapes resulted to have a statistically higher fungal contamination in comparison with the white grapes (*p* ≤ 0.01), with almost double CFU/mL (Table 3). The OTA contamination resulted to increase along with the drying time presenting higher contamination at the final sampling compared to middle and beginning sampling (*p* ≤ 0.01) (Table 3). The highest OTA values were reported at the end of the experiment (21 September 2020), in white grapes dried in tunnel (12.51 ppb), on which the amount of OTA was found to exceed OTA limit of 10 ppb fixed for dried vine fruits (currants, raisins and sultanas). Therefore, OTA must be monitored in dried grapes since drying conditions trigger OTA production and it is mandatory to take it into account during the drying process. The interactions between type of drying and sampling date resulted significant for CFU/mL production (*p* ≤ 0.01), while regarding OTA significantly interactions between grape variety and type of drying were reported (*p* ≤ 0.05) (Table 3). The type of drying influences the drying rate due to higher achieved drying temperatures and consequently the reduced drying duration, which has been found to affect the CFU/mL and OTA production. The grape variety has been also found to favor OTA accumulation in red grape arieties compared to white varieties probably due to the components (i.e., Brix, acidity) of red dried grapes [16,19]. Based on these results, the CFU/mL cannot be used as a standalone criterion for OTA estimation.

**Figure 9:**
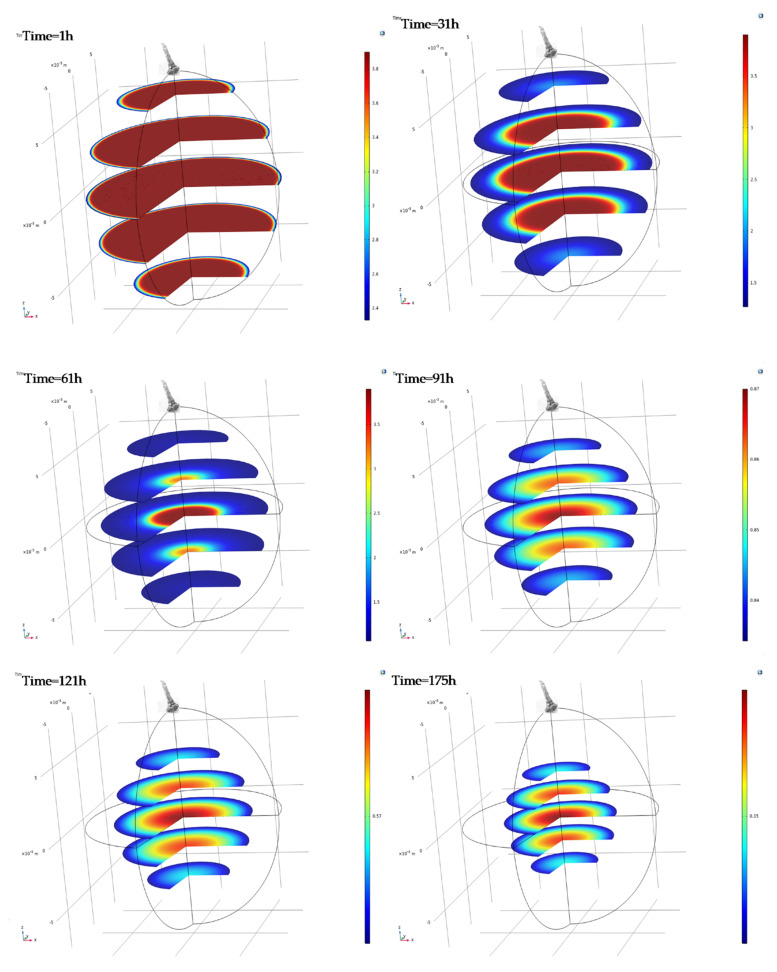
Three–dimensional (3D) slice plots of infected red grapes case during tunnel drying. Plots correspond to 1, 31, 61, 91, 121 and 175 h of drying process. Colour legend box corresponds to the maximum and minimum MC values (kg_water_/kg_dm_) in each plot (ie. for Time = 1 h, colour legend box corresponds to MC = 3.85–2.35 kg_water_/kg_dm_).

In Figure 9 the three–dimensional water content distribution profiles along with the shrinkage effect taking place during tunnel drying of infected red grapes are shown. Profile plots are taken during 1, 31, 61, 91, 121 and 175 h post fungal grape inoculation. The three–dimensional images are drawn rotating the two–dimensional computational domain and the representation of the water content and shrinkage profiles in five distinct levels arranged parallel to the equatorial plane of the grape berry. These images can be used to evaluate the spatio–temporal variations in water content, water content slope towards the grapes surface and the shrinkage during drying, as well the related changes to food quality properties (e.g., bioactive compounds, surface and flesh colour, microbial load, etc.).

The latter function is very useful in building Decision Support Systems (DSS) for the early warning prediction of *A. carbonarius* proliferation and OTA production in table or wine making grapes. Visualization of the three–way interaction among (i) the climate–related abiotic factors, (ii) the biological factors related to grape berry acting as a substrate, and (iii) the fungal population can useful in in–silico experiments where the underling mechanisms of CFU/mL and OTA production can be simulated without the need of time–consuming and labour–intensive research.

The conducted research revealed the relevance of using the grape surface temperature as an indicator to estimate fungal activity during the open air and tunnel solar drying; in fact, the air temperature is significantly different, a fact that can be derived from the computational ambiguity of the drying process and the associated fungal growth and OTA production. Therefore, in future work, the surface temperature of the drying grapes should be monitored throughout the daytime by employing intelligent infrared sensors that can provide real time temperatures. Additionally, the effect of interaction of drying conditions and fungal proliferation on the grape skin, also in relation to its integrity, should be examined, not only through mathematical simulation, but also in terms of electron microscopy. The latter will provide useful information regarding the mechanisms enhancing the fungal proliferation and OTA accumulation. Potential skin disintegration may facilitate the transfer of nutritional compounds from the grapes’ flesh (i.e., sugars) to their outer surface and thus facilitate fungal proliferation and/or OTA production.

## 3. Conclusions

The temperature on the grape surface was on average higher 5–7 °C in tunnel grape berries than in open air–drying grape. The infected grape berries had higher maximum surface temperatures compared to control grape berries. In all the grape berries, surface temperature was higher than air temperature by ≈10 °C. The grapes’ surface temperature was more representative compared to air temperature, during the daylight drying period, which makes this an important indicator of OTA production. The average CFU/mL in open air–drying grapes was significantly higher (*p* ≤ 0.01) than the respective in tunnel drying. On the contrary, the highest levels of OTA were observed in grapes dried in tunnel. Therefore, the CFU cannot be used as a stand–alone indicator of the consequent OTA production. At the middle sampling date (14 September 2020), the a_w_ was below 0.9 in the red and white grapes in tunnel drying due to higher drying temperature and therefore the drying time was shortened, but not in the case of red and white grapes in the open air–drying, where drying process lasted much longer. At this point, it is worth mentioning that the OTA production at 14 September 2020 was 4.04 ppb (in white grapes) and 4.3 ppb (in red grapes) in tunnel drying, which cannot be considered significantly different. On the other hand, significant OTA production was noted at 21 September 2020 in tunnel drying by 85% higher content on average than in the open air–drying, 12.51 ppb (white grapes) and 8.57 ppb (red grapes) when a_w_ was lower than 0.8. This makes a_w_ insufficient as a standalone indicator of OTA production and can be in value only in combination with other indicators.

The grapes D_eff_ in the tunnel drying was higher than the respective D_eff_ in the open air–drying by 54% on average. Partially, this can be explained from the temperature difference which was on average 6 °C higher in the tunnel than in the open air–drying. Comparing the D_eff_ between the infected and control samples per drying case it can be seen that in tunnel drying, the infected grape berries had more than 70% higher D_eff_ than the respective control grape berries (red grapes: 74%, white grapes: 80%). This specific finding is of great importance, since the tunnel dried infected and control grape berries were dried under the same conditions of air temperature and humidity and therefore the noted significant D_eff_ difference could rather be explained by varietal factors affecting the water transport. The peel resistance in the water transfer regarding the tunnel drying grape berries on average was lower than the respective peel resistance in open air–drying. This finding is in line with the drying time which was approximately 390 h for the open air–drying and 220 h for the tunnel drying. Additionally, this is in line with the production of OTA which was significantly higher in tunnel drying than in open air–drying. At this point should it be noted that based on the experimental data, the mechanisms that favor CFU/mL differ from the mechanisms that favor OTA production/accumulation. For example, the shorter drying time in tunnel drying limited the CFU/mL, but on the other hand favored the OTA accumulation, probably due to promoted stress conditions. In tunnel drying, the peel resistance in white control grape berries was 35% higher than in the respective white infected and the red control grape berries was 22% higher than in the respective red infected. The peel resistance in water transport and D_eff_ have been also considered as indicators solving the inverse water diffusion problem. The present results showed that their response is in line with the estimated OTA production something that deserves further investigation.

## 4. Materials and Methods

### 4.1. Plant Material

From 07 September 2020 to 23 September 2020 solar drying experiments were conducted in red (var. *Crimson seedless*) and white (var. *Thompson seedless*) grapes in the open air and in a tunnel to investigate the role of the environmental conditions and drying properties in *A. carbonarius* infection and OTA contamination. For this aim, red and white grapes were purchased from a local market and upon arrival in the laboratory, defective berries (injured, overripe, etc.) were discarded and the remaining were allocated in two lots, the healthy (control) and the infected (infected) grapes; the latter were subjected to artificial inoculation according to the following experimental protocol.

### 4.2. Fungal Inoculum Preparation and Samples Treatment

One strain of *A. carbonarius* (ITEM 5012), previously checked for its ability to produce OTA and stored in the official fungal collection of the Institute of Sciences of Food Production of the National Research Council (ISPA–CNR) in Bari and in the fungal collection of the Department of Sustainable Crop Production (Di.Pro.Ve.S.) of the Università Cattolica del Sacro Cuore in Piacenza, was used for inoculum preparation. The strain was inoculated on Petri dishes containing Potato Dextrose Agar (PDA, Biolife, Milano, Italy) and incubated at 25 °C for 7 days (12 h light photoperiod). At the end of incubation, the dishes were washed with 10 mL of sterile distilled water and the obtained suspension was adjusted to a concentration of 10^4^ conidia/mL. Each sampling group (open air and tunnel drying, white and red grapes bunches) was dipped into the conidial suspension for 5 min and properly allocated for drying. Even untreated grape berries (not inoculated with fungal inoculum) were considered for comparisons and subjected to solar drying until steady weight was achieved. The grape berries were sampled at three sampling times: the beginning of the experiment (07 September 2020), middle of the experiment (14 September 2020) and end of the experiment (21 September 2020). In each of the three sampling times and treatment (four treatments: open air–drying, tunnel drying, white and red infected grapes), three samples consisted of twenty infected grape berries each, (4 treatments × 60 berries = 240 berries) were randomly selected; 3 sampling times × 4 treatments × 60 berries = 720 grape berries were analyzed in total. At each sampling time, infected grape berries were placed in plastic bags and stored at -18 °C until colony forming units’ analysis of fungi (CFU/mL) and OTA quantification (µg/kg) were performed.

### 4.3. Fungal Infection of Grape Berries and Ochratoxin A Detection

Grape berries of the different sampling groups were crushed and must obtained, consisting of the juice and berry residue with the skin included, and this was used for both CFU and OTA analysis. Grape must (1 mL) was added to 9 mL of 1% peptone–water, homogenized with a vortex and the suspension was used for serial dilutions from 10^−1^ to 10^−6^, plated on Potato Dextrose Agar (PDA, Biolife, Milano, Italy) amended with cloramphenicol (0.5%), and incubated at 25 °C for 5 days (12 h light photoperiod); the trial was managed in triplicate. The developed black *Aspergilli* colonies were counted and the results reported as the number of colony forming units per mL of grape must (CFU/mL). For OTA analysis, grape musts were extracted with ethanol (70%) in a ratio 1:1 (v/v) and the mycotoxin concentration levels were determined by the Enzyme–Linked Immunosorbent Assay (ELISA). The sample extracts were analyzed using the AgraQuant *Ochratoxin A* (RomerLabs, Austria) for OTA quantification. Mycotoxin extraction and analysis were performed according to the manufacturer’s instructions and considered reliable as previously determined by Zheng et al. [24].

### 4.4. Hygrothermal Measurements during Drying Experiments

During the solar drying experiments, a hygrothermal sensor and data logger, Hobo U10–003 (Onset Computer Corp., Southern MA, USA) was used to record the air temperature and relative humidity every 10 min, with resolution 0.4 °C and 0.5%, and accuracy ± 0.7 °C and ± 3.0% for temperature and relative humidity, respectively (Figure 1). The daily measurements regarding the surface temperature of grape berries and their weighting were set at 9.30, 14.30 and 18.30, when sun intensity was pronounced. The surface temperature was measured by an infrared temperature thermometer (Kiray 100, KIMO Instruments, France) of accuracy ±2.5 °C (−50 °C to +20 °C), and infrared repeatability ±1.3 °C (−50 °C to +20 °C). The a_w_ was measured by the HygroLab C1 (Rotronic AG, Bassersdorf, Schweiz) equipped with a measurement sensor HC2–AW at 25 °C. The instrument was calibrated in the area of 0.65–0.95 using SCS certified humidity standards EA65–SCS, EA80–SCS and EA95–SCS. Non–linear regression was used to associate the a_w_ with the respective moisture content employing the Statgraphics 19 (Statpoint Technologies, Warrenton, VA, USA) statistical program and the Guggenheim, Anderson and de Boer (G.A.B.) sorption isotherm model (Equation (1)). The M_o_, C, K_b_ parameters are estimated by the Levenberg–Marquardt optimization method,

(1) $$M_{e}=\frac{M_{o}\;C\;K_{b}\;a_{w}}{\left({1-k}_{b}\;a_{w}\right)\left({1-k}_{b}\;a_{w}+C\;K_{b}\;a_{w}\right)}$$

where, M_o_ is the monolayer moisture content; C and K are the adsorption constants, which are related to the energies of interaction between the first and the further sorbed molecules at the individual sorption sites. The weightings were carried out in the eight experimental cases at 20 grape berries per case, placed in aluminium trays (160 grape berries in total). The a_w_ was measured in triplicate in red and white control grapes (tunnel and open air–drying cases) based on a rotation scheme at the end of each experimental day employing the dynamic method. The average initial moisture content of grapes was 4.16 ± 0.1 kg_water_/kg_dm_ (tunnel drying) and 4.51 ± 0.5 kg_water_/kg_dm_ (open air–drying) respectively and was estimated at the end of the experiments gravimetrically at 105 °C for 24 h [25]. The grape drying was terminated when moisture ratio was less than 0.1. The discontinuity in the measurements seen in Figure 1 took place between 248–337 h due to stormy weather where the drying grapes were covered with an opaque plastic sheet.

### 4.5. Seasonal Decomposition Analysis

In order to understand the air temperature and relative humidity daily patterns during the experimental period (7 September 2020–23 September 2020) the recorded data were statistically analyzed by the Statgraphics 19 (Statpoint Technologies, Warrenton, VA, USA) statistical software, applied the seasonal decomposition method which belongs to a family of time series forecasting methods (decomposition models, exponential smoothing models and ARIMA models). The simplest time series forecasting methods use only information on the variable to be forecasted neglecting the factors that affect its behavior. Therefore, these methods extrapolate *trend* and *seasonal patterns*, but they ignore other useful information which is characterized by *irregular* behaviour. The seasonal decomposition analysis divides the time series into three components (*trend–cycle* = the increasing or decreasing value in the series, *seasonality* = the repeating short–term cycle in the series, *irregular or noise* = the random variation in the series) employing *length of seasonality* = 24 h for the seasonal decomposition of the two–time series (air temperature and relative humidity). The *trend–cycle* shows the results of a centred moving average of 24 h length applied to air temperature and relative humidity. The seasonality presents the data divided by the moving average and multiplied by 100. *Seasonal indices* are then computed for each season by averaging the ratios across all observations in that season and scaling the indices so that an average season equals 100. The air temperature and relative humidity data is then divided by the *trend–cycle* and *seasonal estimates* to give the *irregular component* which is multiplied by 100 [26,27].

### 4.6. Data Analysis

The software SPSS (IBM SPSS Statistics ver. 25) was used for statistical data analysis. Analysis of variance (ANOVA) was applied to estimate the mean values of CFU/mL, OTA (μg/kg) detected in infected grapes (red and white grapes) as well as the measured temperatures (air and grape berries’ surface). Tukey’s test was used to identify statistically significant differences between means.

## Figures and Tables

**Table 1:** The geometric mean, min and max values from all the measurements during drying experiments. Homogeneous groups are based on Tukey’s multiple comparison tests.

Measurement Parameter	Drying Method	Infection Status	Grape Type	Min	Max	Geometric Mean	Homogeneous Groups			
Surface Temperature, °C	Tunnel	Infected	Red	32	67	45.5				X
			White	32	62	44.0				X
		Control	Red	33	64	44.0				X
			White	33	63	42.6			X	X
	Open–air	Infected	Red	29	60	38.9		X	X	
			White	30	55	37.4		X		
		Control	Red	30	54	38.6		X	X	
			White	29	51	36.8		X		
Air temperature, °C				15.4	55.1	28.1	X			
Air RH, %				17.4 (0.007 kg_w_/kg_da_)	85.1 (0.017 kg_w_/kg_da_)	41.8 (0.014 kg_w_/kg_da_)				
Experimental a_w_				0.30	0.968	0.68				
Simulated a_w_				0.30	0.975	0.60				

Note: In the parentheses the absolute humidity of air (kg_water_/kg_dry air_) is depicted; Air RH: air relative humidity.

**Table 2:** Estimates of *G.A.B*. sorption isotherm.

Parameter	Estimate	Asymptotic Standard Error	Asymptotic Confidence 95.0%	
			Lower Interval	Upper Interval
M_o_	0.609	4.212	−8.92	10.14
C	0.05067	0.3871	−0.825	0.9263
K_b_	0.9625	0.08855	0.7622	1.163

**Table 3:** Mean values of *A. carbonarius* and OTA in grapes sampled in three different periods (beginning, middle and end of the experiment), in two grape varieties, dried with two different drying methods (open and tunnel). Grape variety, drying type and sampling date were considered as factors in ANOVA.

	CFU (CFU/mL)		OTA (ppb)	
**[1]: Grape Variety**	**		*n.s.*	
Red	3909.48	a	4.53	
White	1154.32	b	4.08	
**[2]: Type of Drying**	**		*n.s.*	
Open–air	4988.43	a	3.50	
In tunnel	75.37	b	5.11	
**[3]: Sampling Point**	*n.s.*		**	
Start	144.31		0.58	b
Middle	3755.56		4.17	a
End	3695.83		8.16	a
**Interactions**				
[1] × [2]	*n.s.*		*	
[1] × [3]	*n.s.*		*n.s.*	
[2] × [3]	**		*n.s.*	
[1] × [2] × [3]	*n.s.*		*n.s.*	

**: *p* ≤ 0.01; *: *p* ≤ 0.05; n.s. = not significant. Different letters mean significant differences according to Tukey test.

**Table 4:** Shrinkage velocities of grapes’ minor and major axis.

Drying Method	Infection Status	Grape Type	Shrinkage Velocity (m/s) Minor Axis (x)	Shrinkage Velocity (m/s) Major Axis (y)
Open air–drying	Control	White	1.418 10^−8^	1.042 10^−8^
		Red	4.844 10^−9^	3.047 10^−9^
	Infected	White	4.683 10^−9^	4.361 10^−9^
		Red	4.511 10^−9^	2.784 10^−9^
Tunnel drying	Control	White	4.747 10^−9^	3.115 10^−9^
		Red	5.230 10^−9^	3.923 10^−9^
	Infected	White	4.472 10^−9^	4.254 10^−9^
		Red	5.734 10^−9^	5.076 10^−9^

**Table 5:** Estimated parameters k_c_, MRE, mean D_eff_ and r_peel_ for all the drying cases.

Drying Method	Infection Status	Grape Type	k_c_ × 10^–9^ (*m*/*s*)	$\bar{T_{s}}$^(1)^ (°C)	r_peel_ × 10^8^ (*s*/*m*)	MRE (%)	$D_{\mathrm{eff}}\times {\;10}^{-10}$ (m^2^/s)
Open air–drying	Control	White	2.75	36.8	3.64	2.17	1.48
		Red	3.33	38.6	3.00	3.64	2.99
	Infected	White	3.16	37.4	3.16	2.55	5.03
		Red	2.61	38.9	3.83	5.53	2.15
Tunnel drying	Control	White	6.14	42.6	1.63	6.20	1.06
		Red	6.37	44.0	1.57	6.74	3.95
	Infected	White	8.31	44.0	1.20	6.72	5.38
		Red	7.79	45.5	1.28	8.30	15.1

**Note: ^(1)^** Geometric means of experimental grape surface temperatures. Used as boundary conditions in the computational simulation.

## Data Availability

Not applicable.
